# ﻿A new species of *Bathypathes* (Cnidaria, Anthozoa, Antipatharia, Schizopathidae) from the Red Sea and its phylogenetic position

**DOI:** 10.3897/zookeys.1116.79846

**Published:** 2022-08-04

**Authors:** Giovanni Chimienti, Tullia Isotta Terraneo, Silvia Vicario, Fabio Marchese, Sam J. Purkis, Ameer Abdulla Eweida, Mattie Rodrigue, Francesca Benzoni

**Affiliations:** 1 Department of Biology, University of Bari Aldo Moro, Bari, Italy University of Bari Aldo Moro Bari Italy; 2 CoNISMa, Rome, Italy CoNISMa Rome Italy; 3 Red Sea Research Center, Division of Biological and Environmental Science and Engineering, King Abdullah University of Science and Technology, Thuwal, Saudi Arabia King Abdullah University of Science and Technology Thuwal Saudi Arabia; 4 University of Milano Bicocca, Milan, Italy University of Milano Bicocca Milan Italy; 5 Center for Carbonate Research, Department of Marine Geosciences, Rosenstiel School of Marine and Atmospheric Science, University of Miami, Miami, FL 33149, USA University of Miami Miami United States of America; 6 Khaled bin Sultan Living Oceans Foundation, Annapolis, U.S.A. Khaled bin Sultan Living Oceans Foundation Annapolis United States of America; 7 Neom, Saudi Arabia Neom Neom Saudi Arabia; 8 OceanX, New York, USA OceanX New York United States of America

**Keywords:** Black corals, cox1-cox3, deep sea, igrN, igrW, Neom, Saudi Arabia

## Abstract

A black coral, *Bathypathesthermophila* Chimienti, **sp. nov.** is described from the Saudi Arabian coasts of the Gulf of Aqaba and north Red Sea (Neom area) using an integrated taxonomic approach. The morphological distinctiveness of the new species is confirmed by molecular analyses. The species thrives in warm and high salinity waters typical of the Red Sea at bathyal depths. It can form colony aggregations on muddy bottoms with scattered, small hard substrates. Colonies are monopodial, feather-like, and attached to a hard substrate through a thorny basal plate. Pinnules are simple, arranged biserially and alternately, and all the same length (up to approximately 20 cm) except for few, proximal ones. Spines are triangular, laterally compressed, subequal, smooth, and simple or rarely bifurcated. Polyps are elongated transversely, 1.5–2.0 mm in transverse diameter. Large colonies can have one or few branches, whose origin is discussed. The phylogenetic position of *B.thermophila* sp. nov. within the order Antipatharia, recovered using three mitochondrial markers, shows that it is nested within the family Schizopathidae. It is close to species in the genera *Parantipathes*, *Lillipathes*, *Alternatipathes*, and *Umbellapathes* rather than to the other available representatives of the genus *Bathypathes*, as currently defined based on morphology. In agreement with previous findings, our results question the evolutionary significance of morphological characters traditionally used to discriminate Antipatharia at higher taxonomic level.

## ﻿Introduction

Black corals (Cnidaria, Anthozoa, Antipatharia) are ubiquitous to the world’s ocean, spanning cold deep to shallow tropical reefs. Traditionally, their description and classification rely on morphological traits, such as the corallum growth form, polyps shape and distribution, number of mesenteries, and size, shape and ornamentation of the skeletal spines (e.g., Opresko 2002; Brugler et al. 2013; Molodtsova and Opresko 2017; Opresko and Molodtsova 2021). With the advent of molecular analyses aiming at the study of phylogenetic relationships and taxa boundaries, several inconsistencies between the phylogenies and the current taxonomy of black corals were revealed. This has led to several Antipatharia families and genera currently recognised as polyphyletic (Brugler et al. 2013; Bo et al. 2018; Barret et al. 2020; Lü et al. 2021). For example, the intergenic spacer between *nad5*-IGR-*nad1* (*igrN*), which is the most variable region of the mitochondrial genome of black corals, in combination with *trnW*-IGR-*nad2* (*igrW*), and the spacer between *cox3* and *cox1* (*cox3-cox1*), have been used to clarify the phylogenetic position and describe new genera and species within the order Antipatharia (Thoma et al. 2009; Brugler et al. 2013; MacIsaac et al. 2013; Horowitz et al. 2020; Opresko and Molodtsova 2021). Moreover, the rRNA internal transcribed spacer region (comprising the 18S, ITS1, ITS2, and 5.8S) has been used in a growing number of publications to elucidate phylogenetic relationships within the families Antipathidae Ehrenberg, 1834, Aphanipathidae Opresko, 2004, and Schizopathidae Brook, 1889 (Lapian et al. 2007; Bo et al. 2018; Barret et al. 2020), as well as in population connectivity studies (Terrana et al. 2021). Finally, the use of next generation sequencing techniques, such as ultra-conserved elements, has recently proven useful in inferring relationships at species level in black corals (Horowitz et al. 2020).

Within the family Schizopathidae, the genus *Bathypathes* Brook, 1889 includes monopodial black corals and currently accounts for 14 valid species (Molodtsova and Opresko 2021; Molodtsova et al. 2022). It occurs in the Pacific, Indian, Atlantic, and Southern oceans, and has been recorded at depths ranging from 102 to 5393 m (Brook 1889; Opresko 2002; Opresko and Molodtsova, 2021; Molodtsova et al. 2022), although its shallowest record (Thomson 1905) is currently considered doubtful. In fact, those of the genus *Bathypathes* are mostly deep-sea species, with few representatives in the mesophotic zone. With deep-sea technologies allowing the exploration of these unseen depths, new *Bathypathes* species have been recently described and, similarly to all the deep-sea antipatharians known thus far, they live in cold waters.

The Red Sea rift is a young ocean basin (Purkis et al. 2012; Augustin et al. 2021) characterised by environmental conditions elsewhere considered extreme for most of the marine fauna. With sea surface temperatures exceeding 32 °C in the summer and deeper water temperatures always higher than 21 °C (Manasrah et al. 2019), the Red Sea arguably provides some of the warmest natural conditions for marine fauna. The basin represents a distinct biogeographic province located at the periphery of the larger Indo-Pacific region, and it is characterised by strong environmental gradients and high endemism of marine organisms (Di Battista et al. 2016; Berumen et al. 2019). Among Red Sea anthozoans, black corals are poorly known except for few museum records (e.g., Brook 1889; Wagner et al. 2012) and spot information about their reproduction and associated fauna (Richmond and Hunter 1990; Herler 2007). The black coral fauna of the Saudi Arabia Red Sea is virtually unstudied from an integrated systematics point of view. This is particularly true in the scantly explored and rapidly developing Neom area, which includes the northern Red Sea Saudi Arabian coast and islands north of Duba, delimited by a narrow coastal strip rapidly descending down to more than 2000 m depth, and the Saudi coast of the Gulf of Aqaba, a 180 km long and 25 km wide semi-enclosed gulf with a NNE-SSW orientation, an average depth of 800 m and a maximum depth of 1852 m. The 2020 OceanX-Neom Red Sea Expedition allowed to explore and assess the marine resources in Neom territory with the ultimate goal to inform Saudi Arabia national conservation strategies. In this context, and with the overall objective to characterise for the first time the Red Sea black coral diversity, a reference collection of Antipatharia including all observed in situ macro-morphologies was obtained. During the benthic surveys, several colonies of monopodial, feather-like black corals were observed from 195 to 688 m depth. Later, morphological study of the sampled material led to their assignation to the genus *Bathypathes* Brook, 1889 (e.g., Brook 1889; Totton 1923; Opresko 2002; Opresko and Molodtsova (2021), thus representing the first record of this genus in the Red Sea.

This study reports the description of a new species of *Bathypathes* found at bathyal depths in the highly saline and warm waters of the Red Sea, representing the first record for the family Schizopathidae in the basin. In particular, *B.thermophila* sp. nov. from the Neom area is described based on traditional morphological characters, and its phylogenetic position within the order Antipatharia is inferred genetically using three mitochondrial loci and including available representatives of all the seven Antipatharia families. This study demonstrates that (a) *B.thermophila* sp. nov. is a new species of Schizopathidae commonly present in the bathyal zone of the Red Sea; (b) black coral diversity in the basin is still poorly known and species new to taxonomy are likely to occur; and (c) traditional morphological characters used to describe genera and species in the order might be subject to convergent evolution and the taxonomy of the order is in need of revision.

## ﻿Materials and methods

### ﻿Sampling and identification

**Figure 1.**
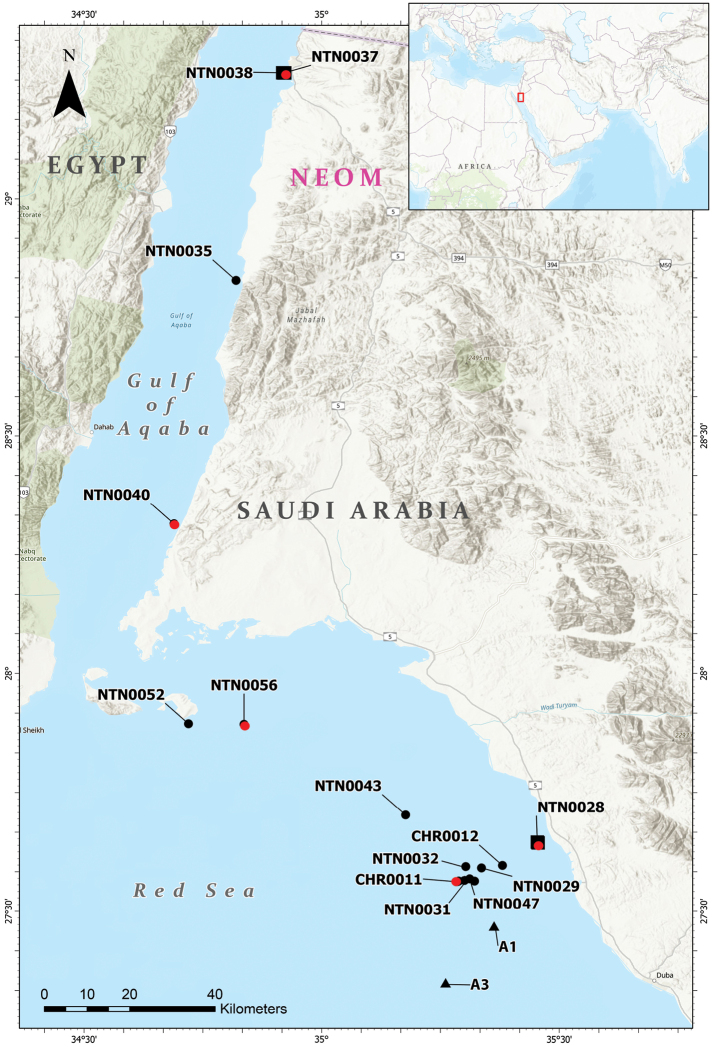
Map of the study area showing the known occurrences of *Bathypathesthermophila* sp. nov. Black dots: observed colonies during the 2020 OceanX-Neom Red Sea Expedition; red dots: sampled colonies; triangles: photographic record by Qurban et al. (2014). Black squares indicate the occurrence of an aggregation of colonies. Codes indicate the submersible (NTN) or remotely operated vehicle (CHR) dives. Coordinates and depth ranges for each dive are reported in Suppl. material 1.

A series of mesophotic and deep-sea explorations were carried out in the Neom area, on the Saudi Arabia coast of the Gulf of Aqaba and northern Red Sea (Fig. 1), from September to November 2020. Sampling occurred during the 2020 OceanX-Neom Red Sea Expedition, aboard M/V “OceanXplorer”, using an Argus Mariner XL108 remotely operated vehicle (ROV) named “Chimaera”, and two Triton 3300/3 submersibles named “Neptune” and “Nadir”. The ROV equipment included a Kongsberg HiPaP 501 Ultra Short Base Line acoustic tracking systems, and a complex light-cameras apparatus with, among the others, one DSPL Super wide-angle CCD camera for landscape view and one HDTV 1080p F/Z colour camera for detailed observations. Each submersible was equipped with a Sonardyne Ranger Pro 2 system and several light and camera systems including a Wide Angle Red DSMC2 Helium 8k Canon CN-E15.5–47 mm lens and a macro Red DSMC2 Helium 8k Nikon ED 70–180mm F4.5-5.6D. Both ROV and submersibles were also equipped with a CTD probe (RBR Maestro CTD and Sea-Bird SBE 19+, respectively), two parallel-aligned scaling lasers providing 100-mm scale and a Schilling T4 hydraulic manipulator for sampling.

Images of living corals were taken in situ with the cameras mounted on the ROV and/or the submersibles. Five colonies were collected at four different localities (Fig. 1). The apical 10-cm portion of the colonies was preserved in 99% ethanol, while the rest of the corallum was air-dried in the shadow for 24 hours. For each collected specimens, macro- and micro-morphological characters were examined and photographed using a Leica M205 A stereomicroscope equipped with a Leica DMC 5004 camera in the Red Sea Research Center laboratory at King Abdullah University of Science and Technology (**KAUST**). Fragments of stem and pinnules were taken from different parts of the dry samples, hydrated in distilled water, cleaned with gentle rinses in diluted sodium hypochlorite (NaClO), then washed with distilled water and dehydrated in a graded ethanol series (Chimienti et al. 2020). Dehydrated skeletal elements were mounted on stubs, coated with a 5-nm thick layer of iridium using a Quorum Q150T S turbomolecular pumped coater, and imaged using a Thermo Fisher Scientific Quattro S Environmental SEM at KAUST Imaging Core Lab.

Type material is deposited at the National Museum of Natural History (**MNHN**), Paris, France. The paratypes are currently kept at KAUST, Thuwal, Saudi Arabia, and at the Zoological Museum of the University of Bari Aldo Moro (**MUZAC**), Bari, Italy.

### ﻿DNA extraction, amplification, and sequence analyses

Total genomic DNA was extracted using the DNeasy Blood and Tissue Kit (Qiagen Inc., Hilden, Germany) following the manufacturer’s protocol. DNA quality and quantity was assessed using a NanoDrop 2000 C spectrophotometer (Thermo Fisher Scientific, Wilmington, USA), and polymerase chain reaction (PCR) was used to amplify three genetic markers. In particular, the mitochondrial *igrN* was amplified with the ND5-5^1^anti10725F-ND1anti11217R primers and the *igrW* with TRPntiF-ND2anti1040R, following Thoma et al. (2009). The primers CO3gen3360F-CO1gen4600R were used for the *cox3-cox1* region following Thoma et al. (2009) and Brugler et al. (2013). All PCRs were carried out in 15 μl reactions using 1X Multiplex PCR Master Mix (Qiagen Inc., Hilden, Germany), purified using Illustra ExoStar (GE Healthcare, Buckinghamshire, UK), and sequenced in forward and reverse direction using an ABI 3730xl DNA Analyzer (Applied Biosystems, Carlsbad, USA) at KAUST Bioscience Core Lab. Chromatograms of the obtained strands were assembled and edited using Sequencher 5.3 (Gene Codes Corp., Ann Arbor, USA). The sequences generated from this study were deposited in GenBank, and accession codes are available in Suppl. material 2.

Sequences of the genus *Leiopathes* Haime, 1849, representative of Leiopathidae Haeckel, 1896, were downloaded from GenBank and used as outgroup in all phylogenetic reconstructions because of its confirmed sister position to the other families of antipatharians (Barret et al. 2020). Newly produced sequences from this study, as well as 157 sequences (*igrN*), 130 sequences of *igrW*, and 67 sequences of *cox3-cox1* retrieved from GenBank, were aligned using MAFFT 7.130b (Katoh and Standley 2013) with the E-INS-i method. The best substitution model for each marker was calculated using PartitionFinder 2 (Lanfear et al. 2017). A Maximum likelihood (ML) phylogenetic reconstruction was performed using RAxML 2 (Stamatakis 2014) on the online CIPRES server (Miller et al. 2012). The ML analyses were run with multiparametric bootstrap analyses of 1000 bootstrap replicates.

## ﻿Results

### ﻿Taxonomy

**Order Antipatharia**

**Family Schizopathidae Brook, 1889**

#### Genus *Bathypathes* Brook, 1889

##### ﻿ Bathypathes thermophila

Taxon classificationAnimaliaAntipathariaSchizopathidae

Chimienti sp. nov.

0DF109C5-A73D-5B3E-89EA-D1C2DD426109

https://zoobank.org/6D09BCE9-D0B6-4DA0-922A-2324A29380B3

Figs 2 , 3 , 4 , 5

###### Material examined.

**Figure 2.**
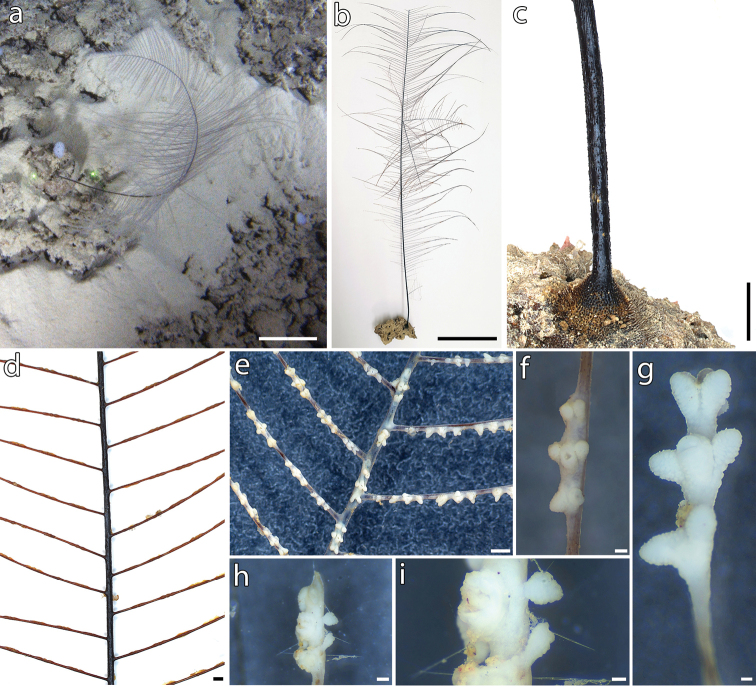
*Bathypathesthermophila* sp. nov., holotype (MNHN-IK-2016-45) **a** colony in situ **b** dry colony **c** base and stem **d** alternate arrangement of pinnules **e** polyps on both stem and pinnules, with **f** detail of a polyp **g** pinnular terminal polyp **h, i** apical polyp of the colony. Scale bars: 10 cm (**a, b**); 5 cm (**c**); 1 mm (**d, e**); 200 µm (**f, h**); 100 µm (**g, i**).

***Holotype***: MNHN-IK-2016-45 (Fig. 2), Red Sea, Duba Channel, 27.643801°N, 35.455505°E, 303 m, 9 Oct 2020, *Neptune* submersible (collection code NTN0028-6). ***Paratypes***: KAUST-NTN0037-8, Red Sea, off Haql (Gulf of Aqaba), 29.264796°N, 34.920449°E, 278 m, 21 Oct 2020, *Neptune* submersible (collection code NTN0037-8); MUZAC-6665, Red Sea, South of Magna (Gulf of Aqaba), 28.31497°N, 34.68936°E, 323 m, 25 Oct 2020, *Neptune* submersible (collection code NTN0040-2); MUZAC-6666, Red Sea, South Sila Island, 27.562322°N, 35.283313°E, 629 m, 12 Oct 2020, *Chimaera*ROV (collection code CHR0011-2); KAUST-NTN0056-3, Red Sea, off Shusha Island, 27.8933°N, 34.83665°E, 597 m, 7 Nov 2020, *Neptune* submersible (collection code NTN0056-3).

###### Diagnosis.

**Figure 3.**
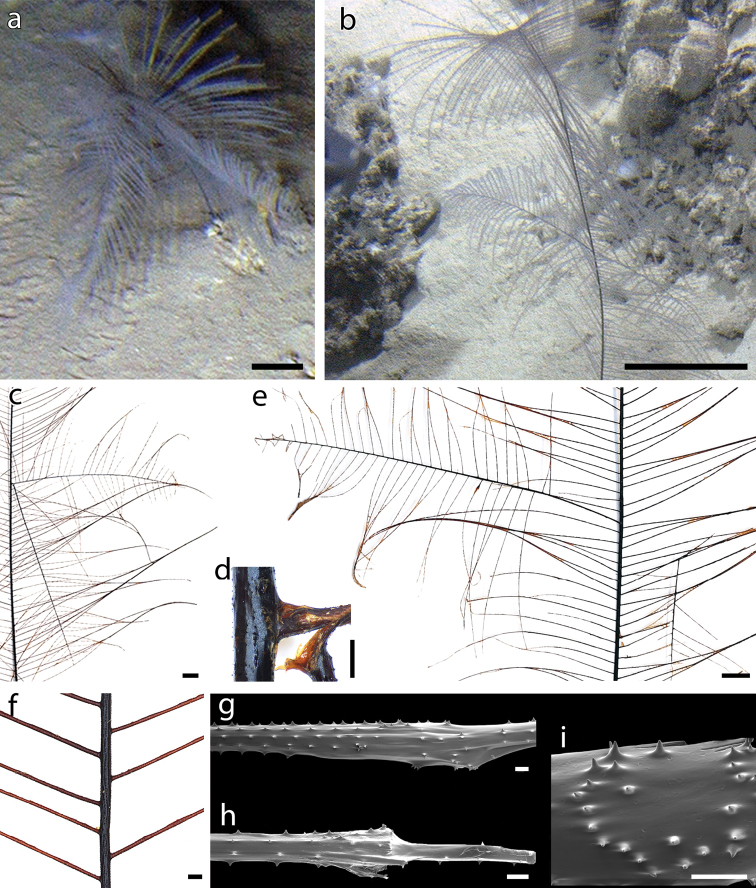
Branching and damages in *Bathypathesthermophila* sp. nov. **a** example of a colony in situ with different branches. Holotype (MNHN-IK-2016-45) **b** ramification observed in situ **c** ramification at the middle of the colony with **d** detail of the ramification. Paratype MUZAC-6665 **e** primary branch (on the left) and secondary branch (on the right) **f** example of two pinnules present on the same side of the stem with respect to the alternate pattern **g** cicatricial nodule on a pinnule **h** narrowing of the pinnular section possibly due to an injury and a subsequent recovery **i** spines arranged irregularly over a skeletal scar. Scale bars: 10 cm (**a, b**); 1 cm (**c, e**); 1 mm (**d, f**); 100 µm (**g–i**).

Colony attached though a basal disk, monopodial, generally unbranched or with a few, random branches, and pinnulate (Figs 2a, b, 3a–e). Stem cylindrical (Fig. 2c), regularly decreasing in diameter from the base to the top. Lower unpinnulated section of the stem (stalk) much shorter than upper pinnulated section. Pinnules simple, up to 20 cm long and subequal in length over most of the corallum, except for proximal and distal ones that are shorter. Pinnules arranged alternately in two lateral rows along the stem (Fig. 2d), although one or few couples of pinnules can be occasionally present on the same side (Fig. 3f). Pinnules inclined of 30° in frontal view (Fig. 2d) and oriented slightly forward in the polypar side with respect to the stem. Pinnular basal diameter 0.18–0.40 mm (0.11 in young colonies). Spacing of pinnules on the same side 2.8–4.3 mm (Fig. 2d), with a pinnular density of 12–18 per 3 cm (up to 20 in young colonies), including pinnules on both sides.

**Figure 4.**
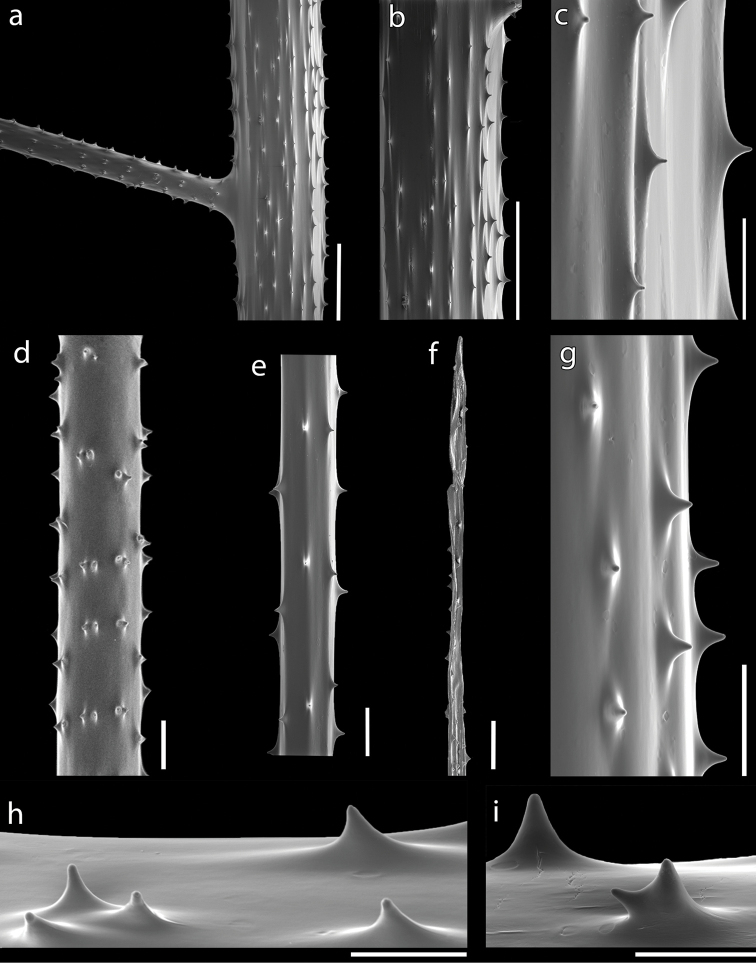
Scanning Electron Microscope of the spines of *Bathypathesthermophila* sp. nov., MNHN-IK-2016-45 (**a–f, h**), MUZAC-6665 (**g, i**) **a** stem and pinnule form the middle portion of the corallum **b** stem from the upper part of the corallum with **c** detail of the spines. Pinnule from the middle portion of the corallum with details of **d** proximal portion **e** distal portion, and **f** terminal portion **g** polypar spines on pinnule **h** row with three polypar spines on a distal pinnule **i** bifurcated polypar spine on pinnule. The right side of vertical skeletal elements and the upper side of horizontal ones is the polypar side. Scale bars: 500 µm (**a, b**); 100 µm (**c–g**); 50 µm (**h, i**).

Spines on the pinnules smooth, laterally compressed, triangular, acute, simple or rarely bifurcated, 0.015–0.025 mm tall (occasionally down to 0.008 mm or up to 0.028 mm), with polypar spines almost the same size of abpolypar ones or slightly larger. Four or five rows of spines in lateral view, each row bearing from one to three spines (Fig. 4d–i), with a density of 5–8 spines per mm (considering double or triple spines as one). Rows can be less obvious on some pinnules.

Spines on the stem smooth, laterally compressed, triangular in profile, acute, simple and enlarged at the base, 0.020–0.030 mm tall. Spines uniformly distributed around the stem, arranged in 10–14 longitudinal rows, each one bearing one to three parallel spines (Fig. 4a–c). Spines often random on the proximal stem and near the basal disk. Basal disk with numerous, elongated spines up to 1.2 mm tall.

Polyps present on one side on both pinnules and stem (Fig. 2e), elongated along the transverse axis, 1.5–2.5 mm in transverse diameter (rarely smaller or larger) and 0.5 mm large (Fig. 2f–i). Polyps sharply divided into three regions, with pairs of tentacles 0.5 mm long (contracted polyps) and 0.2–0.3 mm apart, and prominent oral cones. Polyps distance ranging from 0.5 to 2.5 mm, generally 1 mm, with a density of 10–15 polyps per 3 cm.

###### Description of the holotype.

The holotype (MNHN-IK-2016-45) is a complete colony with an intact basal disk and apex. The colony is attached to the subfossil remains of an irregular echinoid encrusted by various small invertebrates. The colony is 74.5 cm long with a stem basal diameter of 1.8 mm. The unpinnulated stalk is 4 cm long. The basal disk is 7 mm in diameter with numerous, erect and acute spines, up to 0.9 mm tall, all over its surface. The pinnules are simple, bilateral and arranged alternately. Although pinnules are quite flexible, some of them are broken off. The longest remaining ones are ~ 20 cm in length with a basal diameter of ~ 0.28–0.40 mm. The number of pinnules on the corallum is 186 on one side and 181 on the other. Some pinnules are missing at the base of the corallum, likely broken and lost during collection with the sampling manipulator. Within each row, the pinnules are spaced 2.8–3.5 mm apart on the apical portion of the corallum, 3.2–3.7 mm on the middle area and 3.4–4.0 mm on the basal one. The resulting pinnular density is 14 per 3 cm (total for both rows) on the proximal portion of the stem to ~ 16–18 per 3 cm in the median and towards the distal end. The central axial canal is 0.31 mm wide on a pinnule 0.42 mm in diameter, and 0.10 mm on a pinnule 0.22 mm in diameter.

The stem is characterised by six or seven rows of spines in lateral view, although in some areas their distribution can be irregular. A single, 4-cm branch is present in the proximal area of the corallum, and a dichotomous ramification is present in the median area (Fig. 3c, d).

Pinnular spines are small, from 0.017 to 0.022 mm tall, and 0.20–0.31 mm apart. Spines can be double or triple, particularly in the proximal portion of pinnules. Bifurcated spines are also present, although not common.

The polyps are 1.5–2.0 mm in transverse diameter, rarely smaller than 1.5 mm. Polyps density is 11–15 polyps per 3 cm on the stem and 11–13 polyps per 3 cm on the pinnules.

###### Description of the paratypes.

The general morphology of the four paratypes analysed is similar to that of the holotype, with a monopodial corallum and pinnules simple, bilateral and arranged alternately. All paratypes have elongated polyps occurring in a single series on one side of the pinnules and of the stem.

**Figure 5.**
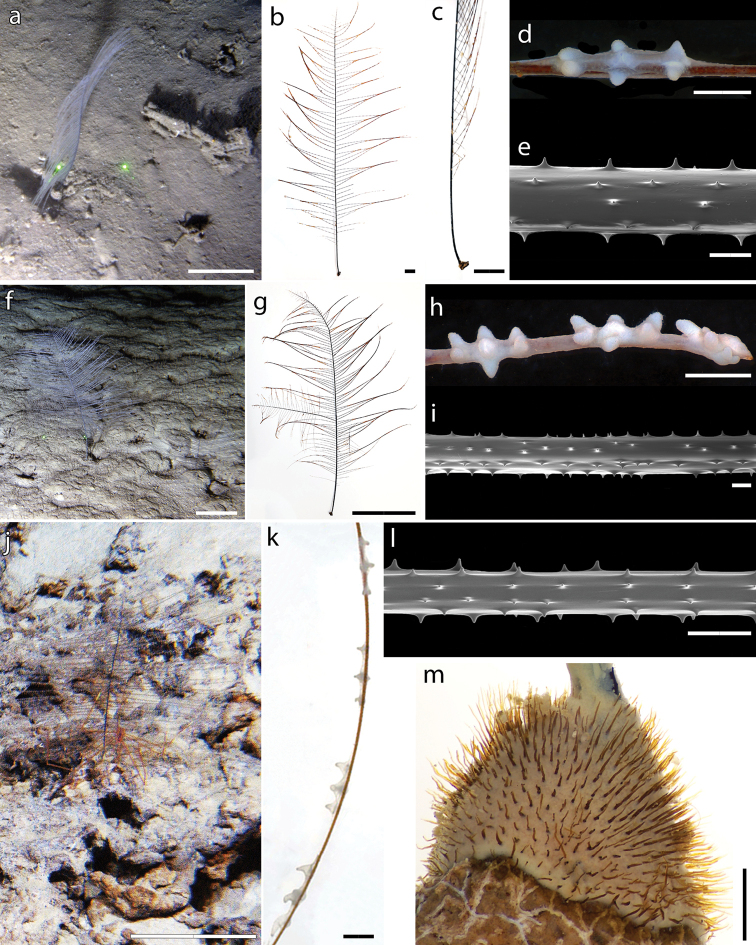
Paratypes of *Bathypathesthermophila* sp. nov. KAUST-NTN0037-8 **a** colony in situ, on a muddy bottom with scattered biogenic hard substrate **b** dry colony in frontal view (polypar side) and in **c** lateral view **d** polyp **e** spines on a proximal pinnule. MUZAC-6665 **f** colony in situ on muddy bottom **g** dry colony in frontal view **h** distal polyps **i** proximal spines on a pinnule from the lower part of the corallum. MUZAC-6666 **j** colony in situ on a hard ground, with a spider crab as epibiont **k** polyps on pinnule **l** spines arrangement on pinnule form the upper part of the corallum **m** base of the colony. Upper side of horizontal skeletal elements is the polypar side. Scale bars: 10 cm (**a, f, g, j**); 1 cm (**b, c**); 1 mm (**d, h, k, m**); 100 µm (**e, i, l**).

Paratype KAUST-NTN0037-8 is a complete colony of 27 cm in length (Fig. 5a, b), with a stem basal diameter of 1 mm. The unpinnulated stalk is 2.8 cm long, while the basal disk is 4 mm in diameter with tall spines, up to 1 mm, erect or curved upwards. The pinnules are up to 9 cm long with a basal diameter of 0.25–0.35 mm and a pinnular density of 14–16 per 3 cm (Fig. 5c). Distance between adjacent pinnules on one side of the stem is 3.3–4.2 mm, except for one couple of pinnules in the median part of the colony that is not alternately arranged. The stem is characterised by six rows of spines in lateral view, although in some areas their distribution is more irregular. Rows are uniformly distributed around the stem, and host spines 0.020–0.028 mm tall. Spines on the pinnules are 0.018–0.022 mm tall, and 0.13–0.20 mm apart (Fig. 5e). Each pinnule bears 4–5 rows of spines in lateral view. Polyps are generally 1.9–2.5 mm in transverse diameter (Fig. 5d), rarely up to 3.0 mm, while young ones can be only 1.3 mm. They are mostly 1 mm apart, although distance can range between 0.8 and 2.3 mm. Density of 10 polyps per 3 cm.

Paratype MUZAC-6665 is a complete colony of 45 cm in length (Fig. 5f, g), with a stem basal diameter of 1.6 mm. The unpinnulated stalk is 3 cm long, while the basal disk is 6.2 mm in diameter with spines up to 1.2 mm tall, erect or curved upwards. Pinnules up to 16 cm long with a basal diameter of 0.30–0.40 mm and a pinnular density of 12–15 per 3 cm. One couple of pinnules at the median and two at the distal part of the stem interrupt the alternate arrangement, being on the same side. A single, primary branch is present on one side of the colony and a single, secondary one on the other side (Fig. 3e). Pinnular spines 0.021–0.028 mm tall, 0.12–0.30 mm apart, and arranged in 4–5 rows in lateral view (Fig. 5i). Spines on the stem arranged in 6–7 rows per side and 0.020–0.030 mm tall. Polyps 2.0–2.5 mm in transverse diameter (occasionally up to 3.0 mm or down to 1.3 mm), with a density of 10–12 polyps per 3 cm (Fig. 5h).

Paratype MUZAC-6666 is a complete colony of 14 cm in length (Fig. 5j), with a stem basal diameter of 0.6 mm. The unpinnulated stalk is 1.6 cm long, while the basal disk is 4 mm in diameter with spines up to 0.8 mm tall (Fig. 5m). The pinnules are up to 7 cm long with a basal diameter of 0.11 mm and a pinnular density of 18–20 per 3 cm. The pinnules are characterised by four or five rows of spines in lateral view, with spines 0.012–0.016 mm tall and 0.10–0.22 mm apart (Fig. 5l). Polyps are 1.5–2.0 mm in transverse diameter, 1.5–2.0 mm apart, with a density of 9–10 polyps per 3 cm (Fig. 5k).

Paratype KAUST-NTN0056-3 is a complete colony 26 cm long, with a stem basal diameter of 0.9 mm. The unpinnulated stalk is 3.0 cm long, while the basal disk is 2.7 mm in diameter with tall spines, erect or curved upwards, up to 0.9 mm tall. Pinnules up to 11 cm long with a basal diameter of 0.18–0.26 mm and a pinnular density of 14–17 per 3 cm. Pinnules are alternate, with adjacent ones spaced 3.2–4.3 mm, except for one, single pair of pinnules present on the same side of the stem, in the median part of the colony. The central axial canal is 0.07 mm in diameter on a pinnule 0.12 mm in diameter. Pinnules with four rows of spines in lateral view, mostly 0.008–0.012 mm tall (some up to 0.018 mm) and 0.10–0.18 mm apart. Stem bearing five rows of spines in lateral view, 0.016–0.020 mm tall. The colony was found dead, without polyps.

###### Etymology.

The species name is derived from the Greek words *thermos* (hot) and *philia* (love, preference for), referring to the occurrence of this species in the rather warm Red Sea waters, especially compared to the water temperatures usually measured in other bodies of water within the same depth range.

###### Distribution.

A colony matching the macro-morphology of *B.thermophila* sp. nov. is shown in Qurban et al. (2014: fig. 3F), where it is reported as an “unidentified sea fan”. It is reported from two areas off Duba (Fig. 1), at 360–720 m depth, extending the known distribution further south from our sampling area. To date, *B.thermophila* sp. nov. is only known from the Gulf of Aqaba and northern Red Sea (Fig. 1), from 195 to 720 m depth.

###### Habitat and ecology.

*Bathypathesthermophila* sp. nov. seems to grow preferentially on scattered hard substrates (e.g., rocks, fresh, fossil or subfossil shells, coral rubble, and subfossil sea urchin tests) surrounded by mud, although it can occur also on rocky bottoms. The holotype (MNHN-IK-2016-45) had settled on a subfossil test of an irregular echinoid, recovered on a sloping rocky substrate covered in a thin muddy layer. The paratypes occurred on muddy bottom with scattered biogenic small substrates (KAUST-NTN0037-8, MUZAC-6665) and on hardground surrounded by mud (MUZAC-6666, KAUST-NTN0056-3).

**Figure 6.**
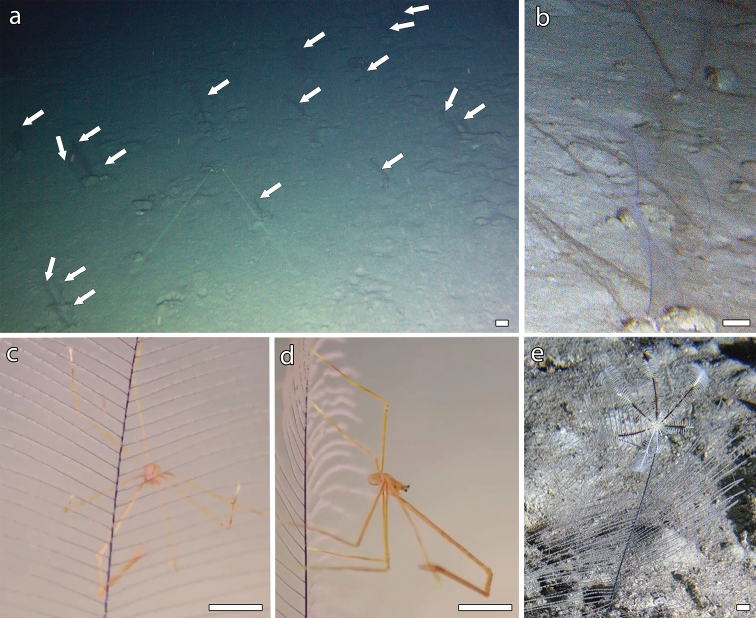
Distribution and associations of *Bathypathesthermophila* sp. nov. **a** aggregations of colonies (white arrows) **b** detail of four co-occurring colonies. Epibionts **c, d** unidentified spider crabs **e** unidentified crinoid. Scale bars: 10 cm (**a, b**); 1 cm (**c–e**).

Water temperature on the seabed was 22 °C, as also reported off Duba by Qurban et al. (2014: fig. 6), indicating that *B.thermophila* sp. nov. lives in relatively warm waters. The species is quite common in the bathyal zone of the Red Sea, where a total of 335 colonies were observed within this study (Suppl. material 1, 2). Aggregations of up to two colonies m^-2^ were found at 280–300 m depth in two different areas (Fig. 1, Suppl. material 1), where *B.thermophila* sp. nov. was the only erect organism (Fig. 6a–d). There, it enhances the benthic structural complexity on flat or gently sloping seabed covered by a mud veneer. *B.thermophila* sp. nov. is often used as habitat by crinoids, spider crabs, and other epibionts (Fig. 6c–e).

###### Comparisons.

Within the genus *Bathypathes*, *B.platycaulus* Totton, 1923 and *B.pseudoalternata* Molodtsova, Opresko and Wagner 2022 show alternate subpinnules. The former is characterised by a peculiar broadening of the stem in the middle region, to which the species name refers (Totton 1923), while *B.thermophila* sp. nov. has a cylindrical stem all along the corallum which lacks a flattened region. *Bathypathesthermophila* sp. nov. also differs from *B.platycaulus* in the pinnular rows of spines (four or five per side homogenously distributed vs. six or seven on one side and four on the other) and in the size of the spines (0.015–0.025 mm vs. 0.040 mm). Finally, the basal diameter of the stem of *B.platycaulus* increases in width in the first 7 cm (from 1.5 mm to 1.75 mm) after which it tapers away gradually up to ~ 0.28 mm at the apex (Totton 1923), being somehow flattered. Conversely, the stem diameter of *B.thermophila* sp. nov. decreases slightly and constantly from the base to the apex.

*Bathypathesthermophila* sp. nov. differs from *B.pseudoalternata* in having a colony which can bear one or a few branches (vs unbranched), higher pinnular density (12–20 vs. 6–12 pinnules per 3 cm), shorter pinnular spines (0.008–0.028 vs 0.030–0.080 mm) with higher density (5–8 vs. 4–5 spines per mm), smaller polyps (1.5–2.5 vs. 3–5 mm in transverse diameter), and higher density of polyps (10–15 vs. 6–7.5 polyps per 3 cm).

###### Remarks.

Large colonies, approximately higher than 40 cm, can show one or few ramifications (Fig. 3a–e). Despite Opresko and Molodtsova (2021) suggesting that monopodial colonies of *Bathypathes* can have one ramification due to damaging events, skeletal analysis in proximity of the branching in *B.thermophila* sp. nov. colonies did not reveal signs of past issues, suggesting that occasional branching can be due to the division of a primary polyp. On the contrary, signs of recovery after mechanical damage were observed on the skeletal of *B.thermophila* sp. nov. without the occurrence of ramifications. These signs were evident mostly as a skeletal swelling (Fig. 3g), a chaotic pattern of spines (Fig. 3i), or a drastic narrowing of the pinnular section (Fig. 3h).

### ﻿Molecular results

A total of four sequences were obtained and analysed for the mitochondrial *igrN* and *igrW*, and *cox3-cox1* region. The final *igrN* alignment consisted of 612 bp, with 60 polymorphic sites, 56 parsimony informative sites, and 72 mutations. The *igrW* alignment consisted of 623 bp, with 93 polymorphic and 69 parsimony-informative sites, and 114 mutations, while the alignment of the *cox3-cox1* was 820 bp long (199 polymorphic and 174 parsimony informative sites, and a total of 250 mutations).

**Figure 7.**
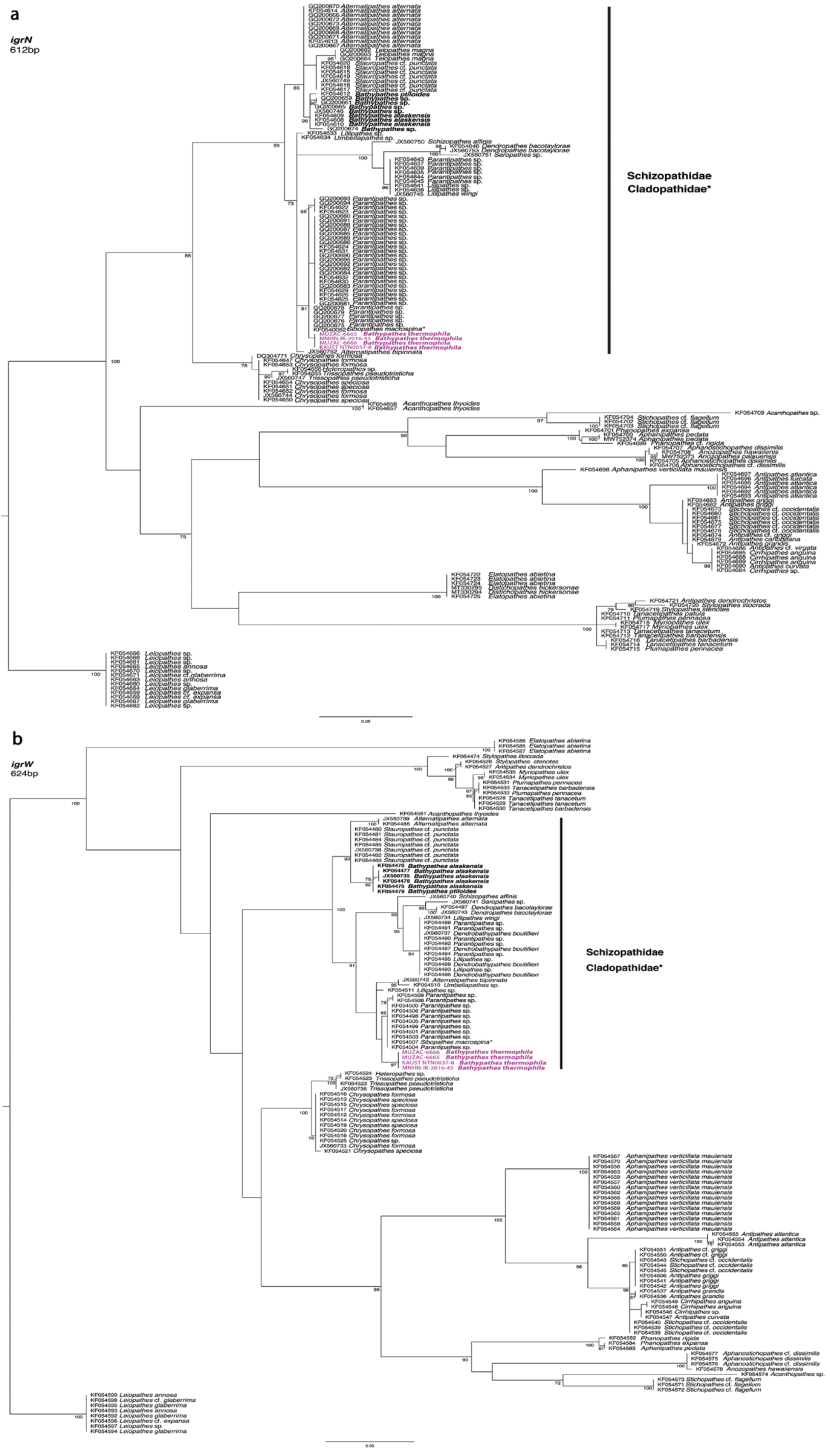
RAxML phylogenetic reconstruction of Antipatharia and the position of *Bathypathesthermophila* sp. nov. (in purple) inferred from two molecular loci **a** the mitochondrial intergenic spacer between *nad5*-igr-*nad1* and **b** the mitochondrial intergenic spacer between *trnW*-igr-*nad2*. Node values are maximum likelihood bootstrap values (> 70%). The genus *Leiopathes* was selected as outgroup. Sequences highlighted by (*) are representatives of the family Cladopathidae.

The *igrN* and *igrW*ML topologies are shown in Fig. 7a, b, with ML bootstrapping support, while the *cox3-cox1* reconstruction is included in Suppl. material 3. *Bathypathesthermophila* sp. nov. is monophyletic, genetically distinct, and molecularly closely related to the available representatives of the genera *Parantipathes* Brook, 1889, *Lillipathes* Opresko, 2002, *Dendropathes* Opresko, 2005, *Saropathes* Opresko, 2002, *Alternatipathes* Molodtsova & Opresko, 2017, and *Umbellapathes* Opresko, 2005 (*igrN*, *igrW*, *cox3-cox1*), all retrieved in the same clade comprising also one sequence of *Schizopathes* Brook, 1889 (*igrN*, *igrW*) and one of *Sibopathes* van Pesch, 1914 (*igrN, igrW*). However, the representatives of *Bathypathes* available in the literature and included in our analyses cluster into a separate clade not closely related to the *B.thermophila* sp. nov. clade. Rather, they are closer to representatives of the genera *Stauropathes* Opresko, 2002 and *Telopathes* MacIsaac & Best, 2013 (Fig. 7a, b; Suppl. material 3).

## ﻿Discussion

Within the Family Schizopathidae, species with a monopodial, unbranched corallum are ascribed to the genera *Bathypathes*, *Schizopathes*, *Abyssopathes* Opresko, 2002, *Saropathes*, *Alternatipathes*, and *Parantipathes*. Our specimens lack the triangular-shaped pinnulated section typical of *Alternatipathes* and *Schizopathes*, the latter also characterised by unattached colonies with a hook-like holdfast for support (Opresko 2002; Molodtsova and Opresko 2017). Moreover, our specimens have only two rows of primary pinnules, simple and lateral, unlike the genera *Abyssopathes* (three rows of pinnules: two lateral and one or more anterior), *Saropathes* (primary pinnules in four rows, with subpinnules) and *Parantipathes* (six or more rows of pinnules) (Opresko 2002).

Based on morphological characters considered diagnostic in black coral taxonomy, we assigned the examined material to the genus *Bathypathes* and described it as a new species. From a molecular point of view, data from three loci, consistently and concordantly placed *B.thermophila* sp. nov. in a clade closely related to the genus *Parantipathes* rather than with the other available sequences of *Bathypathes*. This is relatively unsurprising as previous works already highlighted that the genus *Bathypathes*, as currently defined based on morphological characters, is polyphyletic. For example, rDNA phylogenetic reconstructions showed that *Bathypathes*, comprising representatives of the type species, *B.patula*, clusters with *Stauropathes* (Bo et al. 2018; Lü et al. 2021), and one nuclear sequence of *Bathypathes* sp. (MG023167-YPM IZ 028566) is close to the genus *Telopathes* (Bo et al. 2018; Lü et al. 2021). Given that *Bathypathes, Stauropathes* and *Telopathes* also share some morphological similarities, the morphological characters used to distinguish *Bathypathes* from other genera seem to lack evolutionary meaning (Barret et al. 2020) and further analyses are likely to reveal that *B.thermophila* sp. nov. falls within a different genus. Previous phylogenetic reconstructions based on the same mitochondrial regions have included representatives of *B.patula* (e.g., Brugler et al. 2013; Bilewitch and Tracey 2020). However, some of these samples were recently used by Opresko and Molodtsova (2021) to describe a new species within the genus, *B.alaskensis*, while the sequences from others have never been made available online for comparison. While acknowledging the disagreement between morphological and genetic assignation of the newly described species, in absence of available sequences of the genus *Bathypathes* type species, we decided to adopt a conservative approach and describe *B.thermophila* as a new species within the genus based on traditional morphological characters, still widely used in Antipatharia descriptions.

The overall ML analyses based on the three loci were mostly concordant and recovered a consistent reconstruction of the phylogenetic relationships within the order Antipatharia, in agreement with previously published molecular findings. The three mitochondrial phylogenetic reconstructions obtained consistently placed *B.thermophila* sp. nov. within a distinct molecular lineage including the sequences of all available representatives of the family Schizopathidae and one representative of the family Cladopathidae Kinoshita, 1910 (*Sibopathesmacrospina* Opresko, 1993), with the exception of *cox3-cox1* reconstruction, where no sequences of *Sibopathes* were available (Fig. 7a, b, Suppl. material 3). The inclusion of *S.macrospina* within this molecular lineage is consistent with previous results, since the family Schizopathidae is currently considered polyphyletic only due to the position of *S.macrospina* with the genus *Parantipathes* (Brugler et al. 2013). Other representative genera of the family Cladopathidae (*Trissopathes* Opresko, 2003, *Chrysopathes* Opresko, 2003, *Cladopathes* Brook, 1889, *Heteropathes* Opresko, 2011) are nested in a separate clade. Opresko (1993) already discussed the similarity of skeletal characters between the genera *Parantipathes* and *Sibopathes*, and Brugler et al. (2013) suggested that the morphological characters used to discriminate the latter could be derived. Barret et al. (2020) reconstructed the phylogenetic relationships of black corals on the base of complete mitochondrial genomes, and recovered a concordant topology, with the placement of *S.macrospina* within the Schizopathidae.

Antipatharians are currently largely understudied in the Red Sea, particularly in the mesophotic and aphotic zones. Due to the unique environmental features of this body of water, and its geographic position at the periphery of the greater Indo-Pacific region, a detailed study of Red Sea black corals can greatly contribute to our understanding of the evolution, phylogenetic relationships and biogeography of the whole group, ultimately pushing towards its pending taxonomic revision. Moreover, a reassessment of the morphological characters so far considered informative and diagnostic for black corals taxonomy is needed. In this respect, our molecular reconstructions and their comparison with the observed morphological features represent a first step in this direction and provide a baseline for future works. Future studies of Red Sea black corals will ultimately need to integrate nuclear and Next Generation Sequencing data. Indeed, molecular taxonomy is highlighting similar patterns at different taxonomic levels within the entire order Antipatharia and suggests that a molecular revolution similar to the one which has taken place in the last 20 years for other metazoans in general, and cnidarians in particular, is timely.

## ﻿Conclusions

*Bathypathesthermophila* sp. nov. is described for the Neom region in the north Saudi Arabian Red Sea and represents the first black coral species described from the basin, the first record for the family Schizopathidae, as well as the only member of this family known thus far to live in relatively warm waters at certain depths. Considering the unique ecological and geological features of the Red Sea rift and the peculiar coral fauna that has adapted to live there, it is hardly surprising that the first systematic sampling effort to target black corals diversity and distribution led to the discovery of a new species. Indeed, as the study of the Neom reference collection proceeds, more taxa new to science are likely to be discovered. The study of their phylogenetic relationships and distribution, like in the case of *Bathypathesthermophila* sp. nov., will contribute to our knowledge of black corals and, ultimately, to a revision of their current taxonomic framework, increasingly exposed by molecular investigations to be plagued by polyphyly.

## Supplementary Material

XML Treatment for Bathypathes thermophila

## Supplementary materials

Supplementary material 1 Table S1 Data type Docx file. Explanation note Records of *Bathypathesthermophila* sp. nov. with indication of the dive and the relative gear (CHR: “Chimaera” ROV; NTN: “Neptune” submersible), the geographic coordinates, the depth and the number of colonies observed (N). When several colonies were observed within a transect, coordinates and depth refer to the shallowest and the deepest records. https://binary.pensoft.net/file/723395This dataset is made available under the Open Database License (http://opendatacommons.org/licenses/odbl/1.0/). The Open Database License (ODbL) is a license agreement intended to allow users to freely share, modify, and use this Dataset while maintaining this same freedom for others, provided that the original source and author(s) are credited.Giovanni Chimienti, Tullia Isotta Terraneo, Silvia Vicario, Fabio Marchese, Sam J. Purkis, Ameer Abdulla Eweida, Mattie Rodrigue, Francesca Benzoni

Supplementary material 2 Table S2 Data type Docx file. Explanation note Metadata of the samples of *Bathypathesthermophila* sp. nov. considered in this study. https://binary.pensoft.net/file/723396This dataset is made available under the Open Database License (http://opendatacommons.org/licenses/odbl/1.0/). The Open Database License (ODbL) is a license agreement intended to allow users to freely share, modify, and use this Dataset while maintaining this same freedom for others, provided that the original source and author(s) are credited.Giovanni Chimienti, Tullia Isotta Terraneo, Silvia Vicario, Fabio Marchese, Sam J. Purkis, Ameer Abdulla Eweida, Mattie Rodrigue, Francesca Benzoni

Supplementary material 3 Figure S1 Data type Jpg file. Explanation note RAxML phylogenetic reconstruction of Antipatharia and the position of *Bathypathesthermophila* sp. nov. (in purple) inferred with a cox3-cox1 reconstruction. Node values are maximum likelihood bootstrap values (> 70%). The genus *Leiopathes* was selected as outgroup. Some of the samples reported as *Alternatipathesalternata* are now doubtful with respect to the recently-described *Bathypathespseudoalternata*. https://binary.pensoft.net/file/723397This dataset is made available under the Open Database License (http://opendatacommons.org/licenses/odbl/1.0/). The Open Database License (ODbL) is a license agreement intended to allow users to freely share, modify, and use this Dataset while maintaining this same freedom for others, provided that the original source and author(s) are credited.Giovanni Chimienti, Tullia Isotta Terraneo, Silvia Vicario, Fabio Marchese, Sam J. Purkis, Ameer Abdulla Eweida, Mattie Rodrigue, Francesca Benzoni
