# Supplementary material for: ﻿A new species of Bathypathes (Cnidaria, Anthozoa, Antipatharia, Schizopathidae) from the Red Sea and its phylogenetic position
Source: Zookeys. 2022 Aug 4;1116:1–22. doi: 10.3897/zookeys.1116.79846 (PMC9848741; doi:10.3897/zookeys.1116.79846)
Supplement: Supplementary material 1 — Table S1 [file zookeys-1116-001_article-79846__-s001.docx]

**Supplementary files**

**Table S1** Records of *Bathypathes thermophila* sp. nov. with indication of the dive and the relative gear (CHR: *Chimaera* ROV; NTN: *Neptune* submersible), the geographic coordinates, the depth and the number of colonies observed (N). When several colonies were observed within a transect, coordinates and depth refer to the shallowest and the deepest records.

| **Dive** | **Gear** | **Geographic coordinates** | **Depth (m)** | **N** | **References** |
| --- | --- | --- | --- | --- | --- |
| **CHR0011** | ROV | 27.563665°N, 35.288518°E 27.562311°N, 35.283231°E | 517–630 | 6 | This study |
| **CHR0012** | ROV | 27.596495°N, 35.380516°E | 340 | 1 | This study |
| **NTN0028** | Sub | 27.645578°N, 35.453866°E 27.643801°N, 35.455505°E | 195–303 | 20 | This study |
| **NTN0029** | Sub | 27.596495°N, 35.380516°E | 338 | 1 | This study |
| **NTN0031** | Sub | 27.56503°N, 35.300579°E 27.562746°N, 35.28631°E | 408–603 | 2 | This study |
| **NTN0032** | Sub | 27.594095°N, 35.30333°E | 227 | 1 | This study |
| **NTN0035** | Sub | 34.819861°N, 28.828032°E | 350 | 2 | This study |
| **NTN0037** | Sub | 29.265051°N, 34.922468°E 29.264795°N, 34.920442°E | 250–280 | 153 | This study |
| **NTN0038** | Sub | 29.265327°N, 34.917941°E 29.265257°N, 34.917695°E | 295–300 | 95 | This study |
| **NTN0040** | Sub | 28.31497°N, 34.68936°E | 323 | 1 | This study |
| **NTN0043** | Sub | 27.702827°N, 35.176704°E | 688 | 1 | This study |
| **NTN0047** | Sub | 27.568312°N, 35.311515°E 27.563547°N, 35.321801°E | 248–330 | 32 | This study |
| **NTN0052** | Sub | 27.894453°N, 34.719793°E | 235 | 2 | This study |
| **NTN0056** | Sub | 27.893319°N, 34.836634° E | 597 | 1 | This study |
| **A1** | ROV | 27.470001°N, 35.362778°E | 360–622 | 12 | Qurban et al. 2014 |
| **A3** | ROV | 27.350833°N, 35.260833°E | 654–720 | 5 | Qurban et al. 2014 |
