# Supplementary material for: ﻿A new species of Bathypathes (Cnidaria, Anthozoa, Antipatharia, Schizopathidae) from the Red Sea and its phylogenetic position
Source: Zookeys. 2022 Aug 4;1116:1–22. doi: 10.3897/zookeys.1116.79846 (PMC9848741; doi:10.3897/zookeys.1116.79846)
Supplement: Supplementary material 2 — Table S2 [file zookeys-1116-001_article-79846__-s002.docx]

**Table S2** Metadata of the samples of *Bathypathes thermophila* sp. nov. considered in this study.

| **Sample ID** | **Dive** | **Gear** | **Geographic coordinates** | **Depth (m)** | **Collector** | **GenBank (IgrN)** | **GenBank (IgrW)** | **GenBank (COI)** |
| --- | --- | --- | --- | --- | --- | --- | --- | --- |
| MUZAC-6666 | **CHR0011** | *Chimaera* ROV | 27.562322°N, 35.283313°E | 629 | Giovanni Chimienti | ON759272 | ON759276 | ON759281 |
| MNHN-IK-2016-45 | **NTN0028** | *Neptune* Sub | 27.643801°N, 35.455505°E | 303 | Giovanni Chimienti | ON759275 | ON759278 | ON759283 |
| KAUST-NTN0037-8 | **NTN0037** | *Neptune* Sub | 29.264796°N, 34.920449°E | 278 | Tullia I. Terraneo | ON759273 | ON759277 | ON759280 |
| MUZAC-6665 | **NTN0040** | *Neptune* Sub | 28.31497°N, 34.68936°E | 323 | Francesca Benzoni | ON759274 | ON759279 | ON759282 |
| KAUST-NTN0056-3 | **NTN0056** | *Neptune* Sub | 27.893319°N, 34.836634° E | 597 | Giovanni Chimienti |  |  |  |
