# Supplementary figures and images for: ﻿A new species of Bathypathes (Cnidaria, Anthozoa, Antipatharia, Schizopathidae) from the Red Sea and its phylogenetic position
Source: Zookeys. 2022 Aug 4;1116:1–22. doi: 10.3897/zookeys.1116.79846 (PMC9848741; doi:10.3897/zookeys.1116.79846)

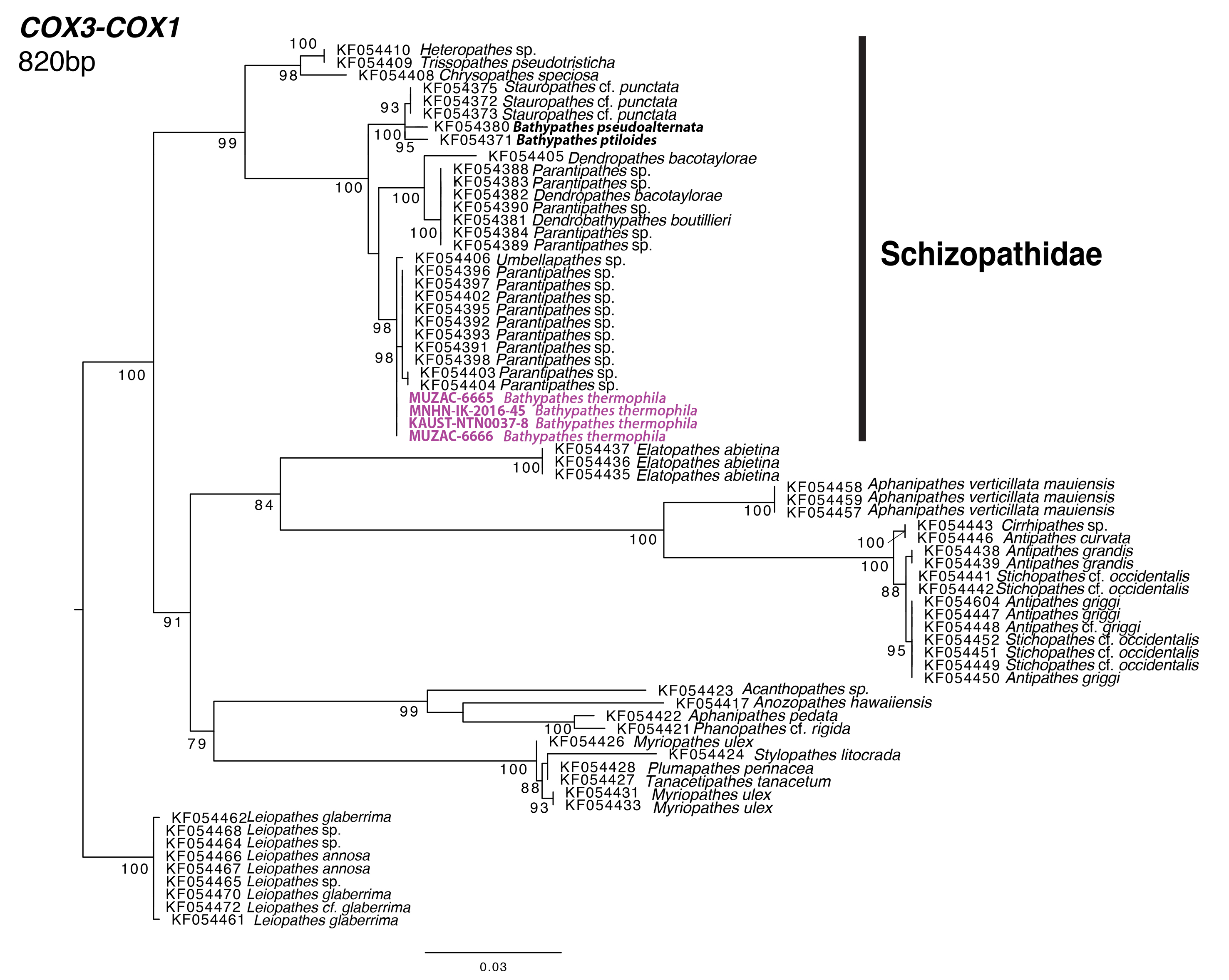

Supplement: Supplementary material 3 — Figure S1 [file zookeys-1116-001_article-79846__-s003.jpg]
